# Genome-wide analysis of *Dof* transcription factors and their response to cold stress in rice (*Oryza sativa* L.)

**DOI:** 10.1186/s12864-021-08104-0

**Published:** 2021-11-06

**Authors:** Jia Liu, Qinglin Meng, Hongtao Xiang, Fengmei Shi, Ligong Ma, Yichu Li, Chunlai Liu, Yu Liu, Baohua Su

**Affiliations:** 1 Institute of Plant Protection, Heilongjiang Academy of Agricultural Sciences, No. 368 Xuefu Road, Nangang District, 150086 Harbin, China; 2 Institute of Farming and Cultivation, Heilongjiang Academy of Agricultural Sciences, 150086 Harbin, China

**Keywords:** Cold, *Dof*, Expression profiling, Rice, Stress response, Transcription factor

## Abstract

**Background:**

Rice (*Oryza sativa* L.) is a food crop for humans worldwide. However, temperature has an effect during the vegetative and reproductive stages. In high-latitude regions where rice is cultivated, cold stress is a major cause of yield loss and plant death. Research has identified a group of plant-specific transcription factors, DNA binding with one zinc fingers (DOFs), with a diverse range of functions, including stress signaling and stress response during plant growth. The aim of this study was to identify *Dof* genes in two rice subspecies, *indica* and *japonica*, and screen for *Dof* genes that may be involved in cold tolerance during plant growth.

**Results:**

A total of 30 rice *Dofs* (*OsDofs*) were identified using bioinformatics and genome-wide analyses and phylogenetically analyzed. The 30 OsDOFs were classified into six subfamilies, and 24 motifs were identified based on protein sequence alignment. The chromosome locations of *OsDofs* were determined and nine gene duplication events were identified. A joint phylogenetic analysis was performed on DOF protein sequences obtained from four monocotyledon species to examine the evolutionary relationship of DOF proteins. Expression profiling of *OsDofs* from two *japonica* cultivars (Longdao5, which is cold-tolerant, and Longjing11, which is cold-sensitive) revealed that *OsDof1* and *OsDof19* are cold-inducible genes. We examined the seed setting rates in *OsDof1*- and *OsDof19*-overexpression and RNAi lines and found that *OsDof1* showed a response to cold stress.

**Conclusions:**

Our investigation identified *OsDof1* as a potential target for genetic breeding of rice with enhanced cold tolerance.

**Supplementary Information:**

The online version contains supplementary material available at 10.1186/s12864-021-08104-0.

## Background

The Asian domesticated rice (*Oryza sativa* L.) is one of the most important crops of the world and serves as a food source for over 60 % of the global population [1]. The two major subspecies of *Oryza sativa* L. are *indica* and *japonica*. *Indica* originates from the eastern part of India and is now cultivated throughout the tropical and subtropical regions of India. *Japonica*, which originated from South China, is now cultivated from North China to Southeast Asia and even as far as West Africa and South America [2].

Because of its tropical or subtropical origin, *Oryza sativa* L. is more sensitive to cold stress than wheat (*Triticum aestivum* L.) and barley (*Hordeum vulgare* L). The optimal temperature for rice germination and seedling growth is 25–30 °C, and germination and final yield can be severely affected at temperatures below 17 °C [3–5]. Cold stress is a major obstacle to rice cultivation in high latitude regions, such as North China, Japan, and South Korea [6]. Furthermore, an annual yield loss of 3–5 million tons of rice in China can be attributed to cold stress.

Cold stress can result from either chilling/freezing (<15 °C) or cold deep-water irrigation (CDWI, 18–19 °C) [7]. Stress due to chilling/freezing usually occurs in the early growth stages and can cause slow germination, developmental delays, and even plant death. The occurrence of cold stress at the early reproductive stage (mostly caused by CDWI) can adversely affect yield and grain quality [8, 9]. In addition to causing physical damage, cold stress also triggers physiological and metabolic changes in rice plants. For example, low chlorophyll production and decline in photosynthetic efficiency have been observed in rice plants due to cold stress [10, 11]. The induction of reaction oxygen species (ROS) and malondialdehyde (MDA) triggered by cold stress provokes cellular oxidative damage and metabolic malfunctioning, ultimately disturbing the lipid composition of cell membranes [12, 13]. The accumulation of amino acid proline and soluble sugars in plant cells has been reported to function in the natural defense against cold stress [14, 15]. An elevation in the levels of antioxidants in plants can help in scavenging ROS and restoring cellular functions. Overexpression of *OsAPXa*, which encodes an ascorbate peroxidase, was found to enhance chilling tolerance at the booting stage in rice plants [16].

The molecular mechanisms by which rice plants sense and respond to cold stress have been intensively investigated. Three pathways involved in the response to cold stress include abscisic acid (ABA) signaling, the dehydration-responsive element-binding protein 1 s/C-repeat-binding factors (DREB-CRT/DRE) pathway, and the mitogen-activated protein kinase (MAPK) cascade [13, 17–20]. Transcription factors (TFs) play key role in transmitting these pathways. For example, upon cold stress, the NAC-type transcription factor *OsNAC6* is upregulated and consequently activates a peroxidase, an antioxidant enzyme [13]. Similarly, *OsNAC5* is induced by cold stress, and transgenic lines with OsNAC5 overexpression show high tolerance to salinity [21]. In addition, cold stress induces the expression of *OsDREB1A* and *OsDREB1B* in rice, which are members of the TF family of DREB1s/CBFs [22–24]. Thus, TFs are pivotal nodes in the signaling networks associated with cold sensing and responding in rice plants.

The DNA binding with one zinc finger (DOF) family of transcription factors is a plant-specific family, and genome-wide identification of *Dofs* has been reported for many species, including *Arabidopsis* and crops such as oilseeds rape, wheat, and cotton [25–28]. This gene family has not been detected in *Gongylonema pulchrum*, *Drosophila melanogaster*, or *Saccharomyces cerevisiae*. The DOF TFs play an important role in diverse physiological processes of plants, including vegetative and reproductive development, stress sensing and response, and circadian cycles [29–34]. DOF are approximately 200–400 amino acids in length and contain a highly conserved Dof domain at the N-terminus and a transcriptional regulation domain at the C-terminus [25, 35–37]. The Cys2/Cys2 zinc finger in the Dof domain is essential for recognizing the core sequence of 5ʹ-(AT)/AAAG-3ʹ in genomic regions upstream of target genes [38, 39].

The involvement of DOFs in biotic/abiotic stress responses has been described in several plant species, including rice [27, 40–44]. Zhou et al. investigated ABA, salt, and PEG treatments in rice seedlings and found that 27 *OsDofs* were either induced or suppressed by at least two of the treatments [42]. However, whether *OsDof* genes participate in cold stress responses in rice has not been investigated.

Here we used bioinformatics tools to investigate *Dof* family genes in two subspecies of rice. We further identified *OsDof1* as a cold-inducible *Dof* gene and demonstrated that the expression level of *OsDof1* in a cold-sensitive rice cultivar was positively correlated with tolerance and performance under cold stress. These results suggest the potential of *OsDof1* as a bioengineering target for improved cold tolerance in rice.

## Results

### Genome-wide identification and analysis of *Dof* genes

**Fig. 1:**
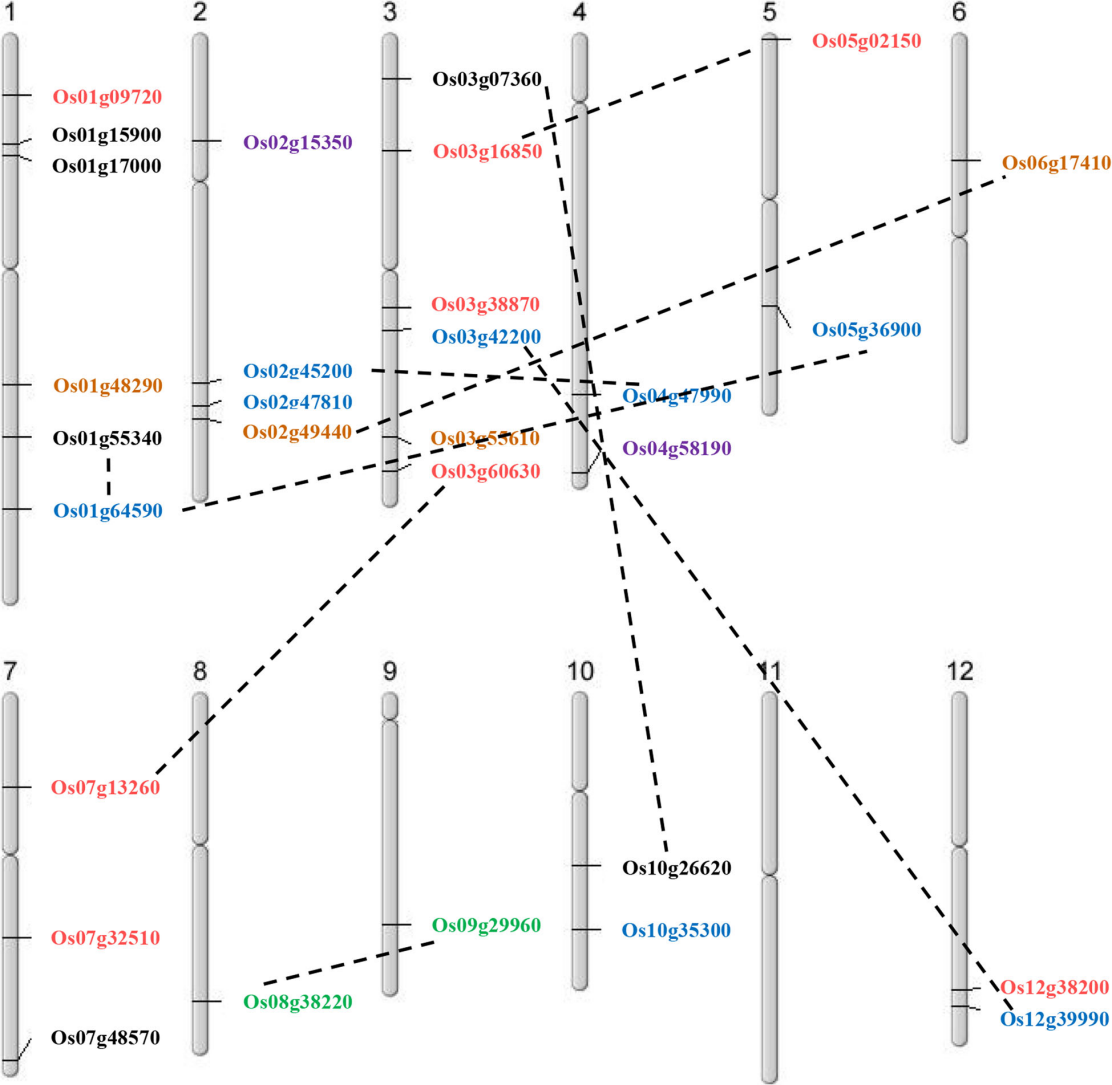
Chromosomal locations and duplication events of rice *Dof* genes. In the phylogenetic tree, *OsDof* genes are differentiated by colors based on their subfamilies. The chromosome number is indicated. Linking of nine pairs of potential duplicated genes is shown with dashed lines

**Fig. 2:**
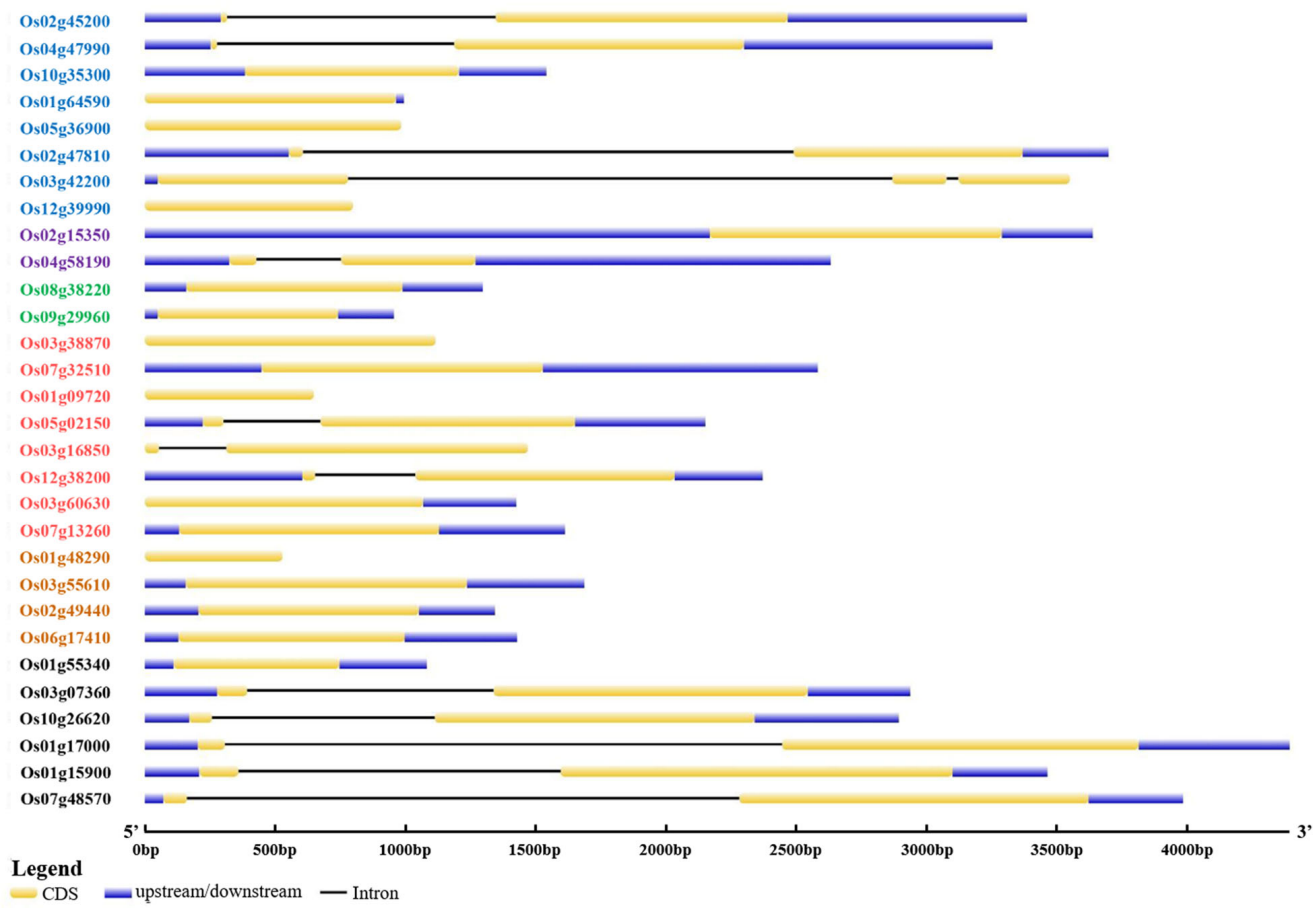
Genetic structure of rice *OsDof* genes

We analyzed the publicly available genome sequences of *japonica* and *indica* and identified 30 non-redundant *OsDofs*, 27 of which have identical sequences between the two subspecies. The *OsDof* genes were located on 11 out of 12 chromosomes (excluding chromosome 11), with chromosomes 1 and 3 having the largest number of *OsDofs* (Fig. 1). The gene length of *OsDofs* varies from 528 to 2583 base pairs. Among the 30 genes, 17 (53.3 %) were single-exon genes and 12 (30 %) had one intron (Fig. 2). Alternative splicing isoforms exist in *OsDof8*, *OsDof17*, *OsDof18*, and *OsDof28*. All 30 *OsDofs* harbor a Dof domain, which contains a highly conserved zinc finger with four cysteines (Table 1).

**Table 1:** Highly conserved Dof domains among the 30 identified rice DOFs

OsDOF1	CPRCDSPNTKFCYYNNYSLSQPRYFCKGCRRYWTKGGSYRNVPVGGGCRK
OsDOF2	**CPRC**N**S**MD**TKFCY**Y**NNY**NVN**QPR**HF**C**KN**C**Q**R**Y**WT**A**GG**TM**R**NVPVGAGRRK
OsDOF3	**CPRC**E**S**TN**TKFCY**Y**NNY**NLA**QPR**HF**C**KA**C**R**R**Y**WT**R**GG**AL**R**NLPVGAGTRN
OsDOF4	**CPRC**N**S**ME**TKFCY**F**NNY**NVH**QPR**HF**C**RN**C**Q**R**Y**WT**A**GG**AM**R**NVPVGAGRRR
OsDOF5	**CPRC**G**S**RE**TKFCY**F**NNY**NVR**QPR**HL**C**RS**C**R**R**Y**WT**A**GG**AL**R**NVPVGPGRRK
OsDOF6	**CPRC**R**S**RD**TKFCY**F**NNY**NVN**QPR**HF**C**KA**C**H**R**Y**WT**A**GG**AL**R**NVPVGAGRRK
OsDOF7	**CPRC**E**S**TH**TKFCY**Y**NNY**SLS**QPR**YF**C**KT**C**R**R**Y**WT**K**GG**SL**R**NVPVGGGCRK
OsDOF8	**CPRC**E**S**TN**TKFCY**Y**NNY**NLS**QPR**HF**C**KS**C**R**R**Y**WT**K**GG**VL**R**NLPVGGGCRK
OsDOF9	**CPRC**E**S**TH**TKFCY**Y**NNY**SLS**QPR**YF**C**KT**C**R**R**Y**WT**K**GG**SL**R**NVPVGGGCRK
OsDOF10	**CPRC**N**S**TN**TKFCY**Y**NNY**SLQ**QPR**YF**C**KT**C**R**R**Y**WT**E**GG**SL**R**NVPVGGGSRK
OsDOF11	**CPRC**D**S**SN**TKFCY**Y**NNY**NLT**QPR**HF**C**KT**C**R**R**Y**WT**K**GG**AL**R**NVPIGGGCRK
OsDOF12	**CPRC**S**S**MD**TKFCY**Y**NNY**SLS**QPR**HF**C**KT**C**R**R**Y**WT**K**GG**AM**R**NLPVGAGRRK
OsDOF13	**CPRC**D**S**TD**TKFCY**Y**NNY**SLS**QPR**YF**C**KT**C**R**R**Y**WT**K**GG**AL**R**NLPVGGGGRR
OsDOF14	**CPRC**D**S**TN**TKFCY**Y**NNY**SLS**QPR**HF**C**KT**C**R**R**Y**WT**R**GG**SL**R**NLPVGGGCRR
OsDOF15	**CPRC**N**S**SN**TKFCY**Y**NNY**NLT**QPR**HF**C**KT**C**R**R**Y**WT**K**GG**AL**R**NVPIGGGCRK
OsDOF16	**CPRC**E**S**TN**TKFCY**F**NNY**SLS**QPR**HF**C**KT**C**R**R**Y**WT**R**GG**AL**R**NLPVGGGCRR
OsDOF17	**CPRC**N**S**SN**TKFCY**Y**NNY**NLT**QPR**YF**C**KT**C**R**R**Y**WT**K**GG**AL**R**NVPIGAGCRK
OsDOF18	**CPRC**N**S**TN**TKFCY**Y**NNY**SLQ**QPR**YF**C**KT**C**R**R**Y**WT**E**GG**SL**R**NVPVGGGSRK
OsDOF19	**CPRC**D**S**TN**TKFCY**F**NNY**SLS**QPR**HF**C**RA**C**R**R**Y**WT**R**GG**AL**R**NLPVGGGYRR
OsDOF20	**CPRC**D**S**SN**TKFCY**Y**NNY**NLS**QPR**HF**C**KA**C**R**R**Y**WT**K**GG**LL**R**NVPVGGGCLK
OsDOF21	**CPRC**D**S**TN**TKFCY**F**NNY**SLT**QPR**HF**C**KA**C**R**R**Y**WT**R**GG**AL**R**NLPVGGGFRR
OsDOF22	**CPRC**D**S**AN**TKFCY**F**NNY**SLS**QPR**HF**C**KA**C**R**R**Y**WT**R**GG**TL**R**NLPVGGGCRK
OsDOF23	**CPRC**N**S**MD**TKFCY**Y**NNY**NIN**QPR**HF**C**KS**C**Q**R**Y**WT**A**GG**SM**R**NLPVGAGRRK
OsDOF24	**CPRC**D**S**TN**TKFCY**Y**NNY**SLS**QPR**HF**C**KS**C**R**R**Y**WT**K**GG**LL**R**NVPVGGGTRK
OsDOF25	**CPRC**D**S**TN**TKFCY**F**NNY**SLS**QPR**HF**C**KA**C**R**R**Y**WT**R**GG**AL**R**NLPVGGGFRR
OsDOF26	**CPRC**S**S**MD**TKFCY**F**NNY**NVN**QPR**HF**C**KH**C**Q**R**Y**WT**A**GG**AM**R**NVPVGAGRRK
OsDOF27	**CPRC**D**S**SN**TKFCY**Y**NNY**NLS**QPR**HF**C**KA**C**R**R**Y**WT**K**GG**LL**R**NVPVGGASRR
OsDOF28	**CPRC**D**S**TN**TKFCY**F**NNY**SLS**QPR**HF**C**KA**C**R**R**Y**WT**R**GG**GC**R**NLPVGGGCRR
OsDOF29	**CPRC**E**S**PN**TKFCY**Y**NNY**SLS**QPR**YF**C**KG**C**R**R**Y**WT**K**GG**SL**R**NVPVGGGCRK
OsDOF30	**CPRC**R**S**TN**TKFCY**Y**NNY**NTA**QPR**HF**C**RA**C**R**R**Y**WT**H**GG**TL**R**NLPVGGGCRR

Multiple sequence alignment of Dof domains from 30 OsDOFs was performed using Clustalx1.87. Conserved amino acid residues in the Dof domains are shown in blue, with the conserved four cysteine residues highlighted in yellow

### Motif location and phylogenetic relationship of OsDOFs

**Fig. 3:**
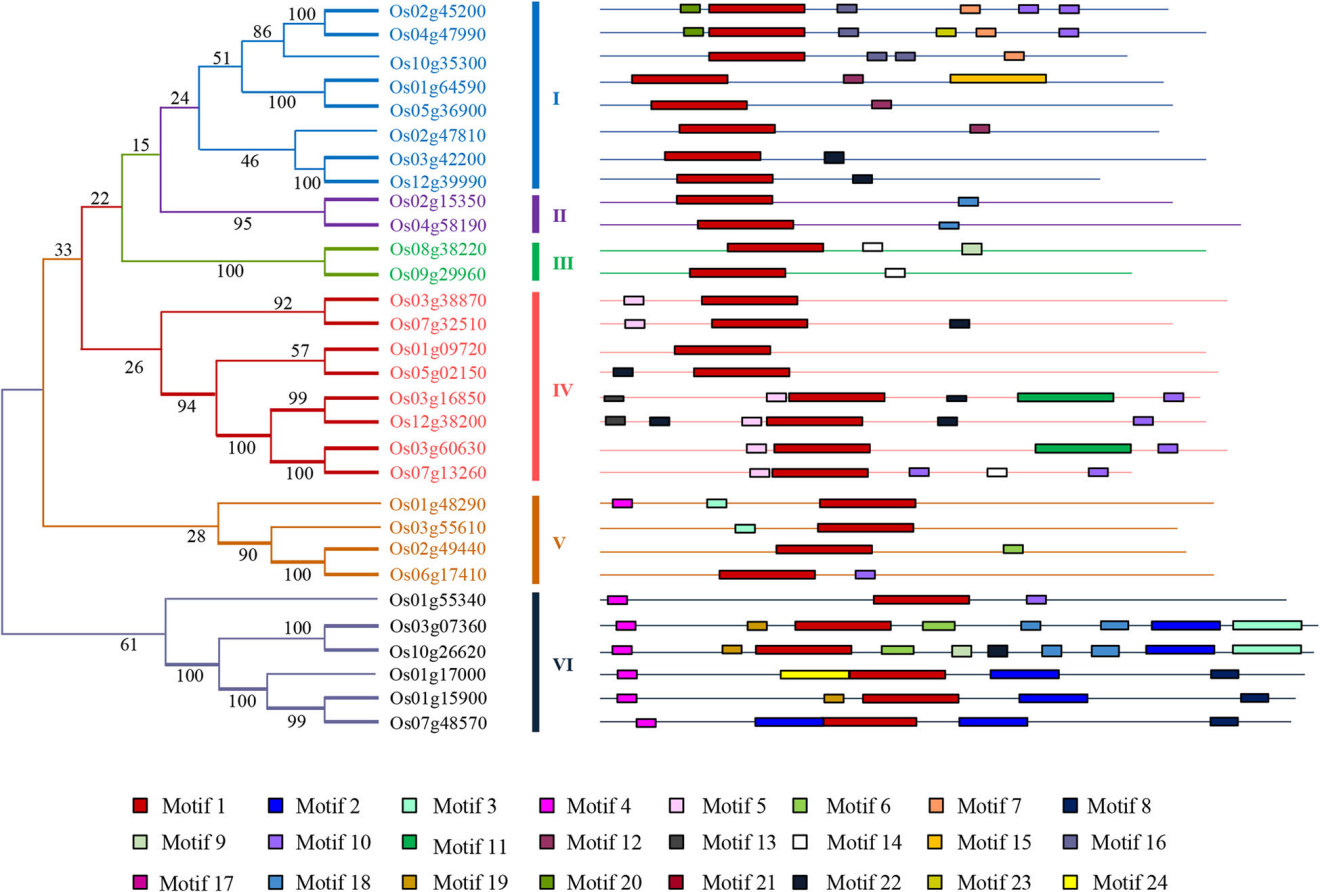
Phylogenetic analysis and common domain identification of 30 DOFs. Left: A phylogenetic tree was constructed based on the alignment of 30 OsDOFs in MEGA 6.0 with a bootstrap replicate value set as 1000. Percentage bootstrap scores are indicated on the nodes. Six subfamilies are designated (I–VI). Right: A total of 24 common motifs were identified among the 30 OsDOFs. Striped boxes with different colors are used to represent the domains (see Supplemental Table 1 for the sequence of each domain). Motif 1 is the highly conserved Dof domain

OsDOFs were classified into six groups (I–VI) based on the structure of Dof domain, with the largest number of OsDOFs (eight) in subfamilies I and IV and the lowest number (two) in subfamilies II and III (Fig. 3). A total of 24 protein motifs were identified in the DOF family, including the conserved Dof domain (Motif 1) that was present in all OsDOFs (Supplemental Table 1). Some DOFs showed unique motifs. For example, motifs 2 and 4 were unique in five members of subfamily VI (Os01g15900, Os07g48570, Os01g17000, Os03g07360, and Os10g26620). In addition, Os01g15900, Os07g48570, and Os01g17000 have a unique motif (motif 22) at the C terminus, providing a clue for their phylogenetic closeness (Fig. 3). Motif 5 is unique to subfamily IV, and only two members of subfamily V contain motif 3. Diversity in the C terminus of OsDOFs was observed, which was highly associated with the phylogenetic closeness among OsDOFs.

### Phylogenetic comparison of DOFs from multiple species

**Fig. 4:**
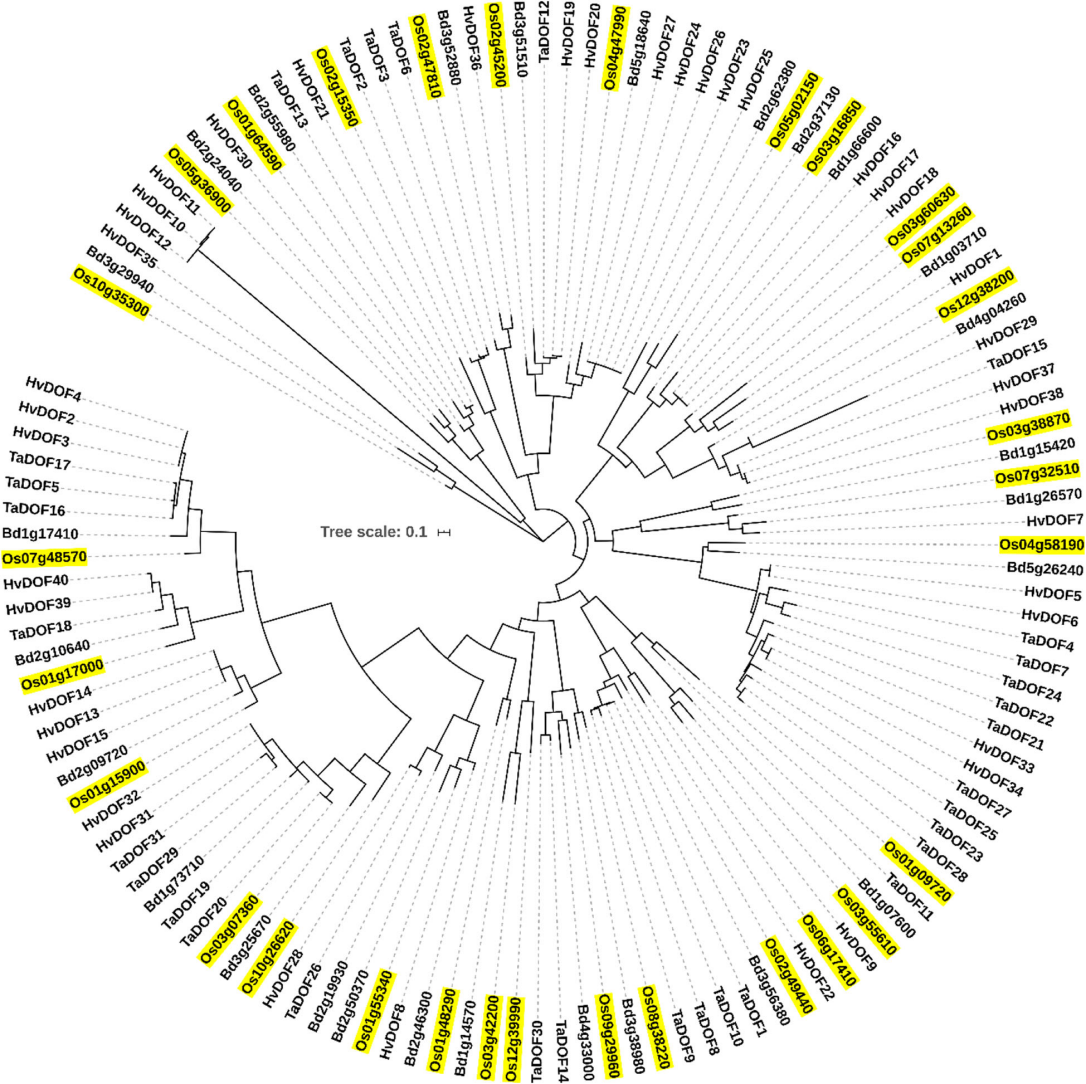
Phylogenetic tree of DOF proteins from barley, wheat, stiff brome, and rice. Full-length protein sequences were aligned using ClustalX, and a joint phylogenetic tree was generated in MEGA 6.0 using the maximum likelihood method. Rice DOFs are indicated in yellow

We performed phylogenetic investigations on DOF proteins from four monocotyledon plants: barley (*H. vulgare*, 40 DOFs), wheat (*T. aestivum*, 31 DOFs), stiff brome (*B. distachyon*, 27 DOFs), and rice (30 DOFs) [45–47]. The maximum likelihood method and the Jones-Taylor-Thornton (JTT) matrix-based model were used to evaluate the evolutionary history [48]. Based on the 128 amino acid sequences, a joint phylogenetic tree was generated (Fig. 4). A phylogenetic tree based on DOF proteins obtained from the abovementioned four monocotyledon plants and the model plant species *Arabidopsis* is presented in Supplemental Fig. 1. Putative orthologues were identified for the majority of rice DOFs (for example, Os03g55610 (OsDOF15) with paralogues HvDOF9, TaDOF11, and Bd1g07600 from other species). These findings indicate a close evolutionary relationship among the DOF family proteins across the investigated monocot species. Our analysis revealed nine duplication events among the rice *OsDofs* (Fig. 1). These potential duplications, which are proposed interchromosomal events, occurred within all subfamilies except for subfamily II, which only contains two genes. No tandem duplication of rice *Dof* genes was observed.

### Expression of *OsDof* genes in different tissues under cold treatment

**Fig. 5:**
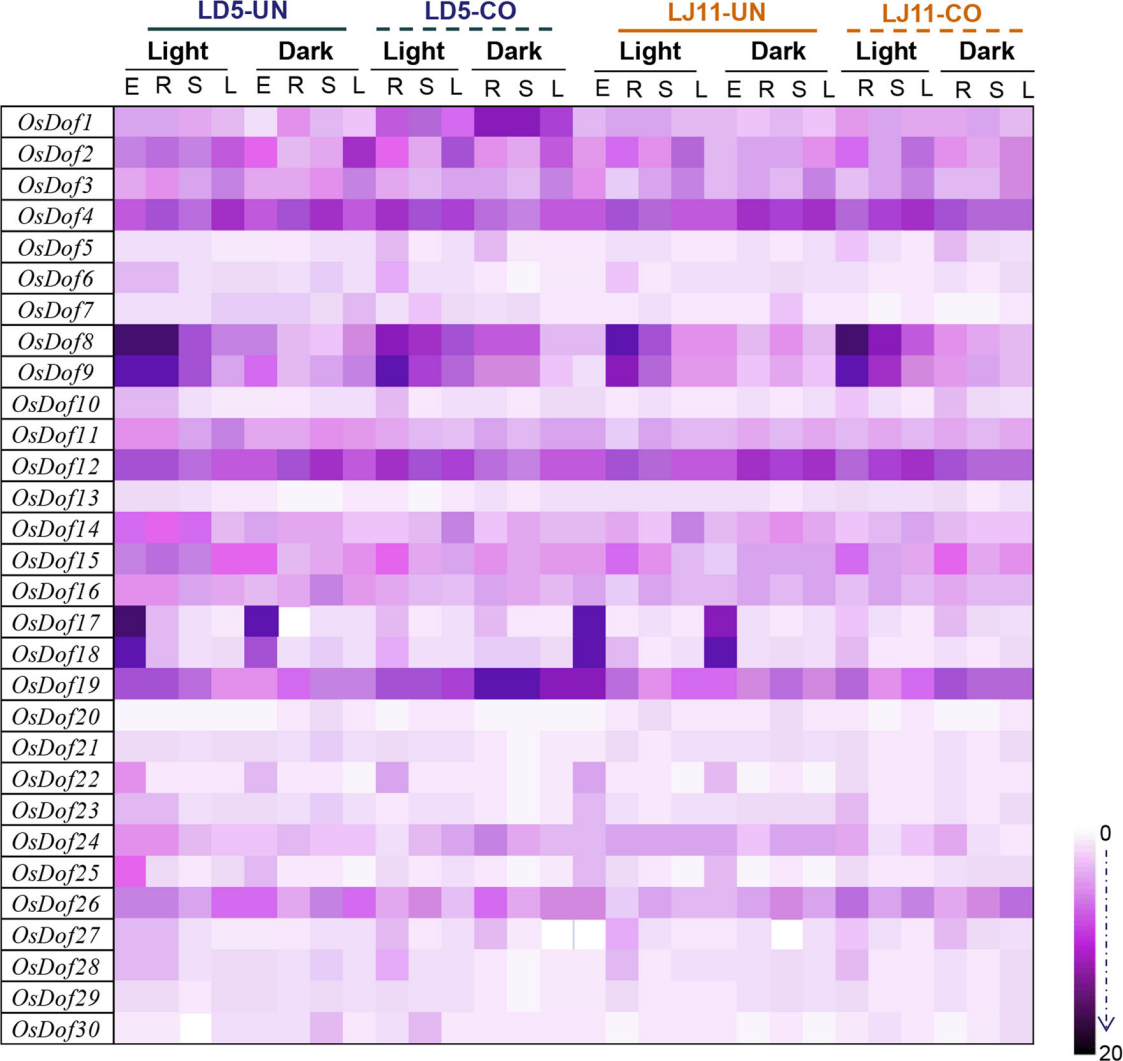
Expression of *OsDof* genes from different tissues in two rice cultivars, LD5 and LJ11, under cold treatment. Expression profiling was performed using qRT-PCR assay with rice *OsActin1* as an internal standard. Three biological replicates were performed for each sample, each with three technical replicates (Supplemental Table 3). UN: untreated plants grown under natural conditions; CO: cold-treated plants. Treatment was performed under both light and dark conditions. E: seeds; R: roots; S: stems; L: leaves. Color scale indicates relative levels of expression

We next examined the expression of the 30 *OsDofs* in cold-tolerant LD5 and cold-sensitive LJ11 plants by qRT-PCR using primers listed in Supplemental Table 2. Various tissues, including leaf, stem, root, and seeds were investigated (Fig. 5 and Supplemental Table 3). Tissues were sampled from plants grown under long day condition. We identified 9 gene members with low expression levels (relative intensity 0–5, including *OsDof5*, *OsDof6*, *OsDof7*, *OsDof10*, *OsDof13*, *OsDof20*, *OsDof21*, *OsDof29*, and *OsDof30*), 13 gene members with medium expression levels (relative intensity 5–10, including *OsDof1*, *OsDof2*, *OsDof3*, *OsDof11*, *OsDof14*, *OsDof15*, *OsDof16*, *OsDof22*, *OsDof23*, *OsDof24*, *OsDof25*, *OsDof27*, and *OsDof28*), and 8 gene members with high expression levels (relative intensity 10–20, including *OsDof4*, *OsDof8*, *OsDof9*, *OsDof12*, *OsDof17*, *OsDof18*, *OsDof19*, and *OsDof26*).

Most *OsDofs* showed tissue-specific expression. For example, *OsDof17*, *OsDof18*, *OsDof22*, and *OsDof25* were seed-specific; *OsDof8*, *OsDof9*, *OsDof27*, and *OsDof28* were preferentially expressed in roots; and *OsDof2*, *OsDof3*, *OsDof11*, and *OsDof14* were highly expressed in leaves.

Six genes appeared to be regulated by cold treatment at booting stage and dark treatment: *OsDof1*, *OsDof8*, *OsDof9*, *OsDof17*, *OsDof18*, and *OsDof19* (Supplemental Table 3). Only two genes, *OsDof1* and *OsDof19*, were upregulated by cold treatment in all investigated tissues of the cold-tolerant LD5 plant (Supplemental Fig. 2), suggesting their potential roles in cold tolerance.

### *OsDof1* contributes to cold tolerance in rice

**Fig. 6:**
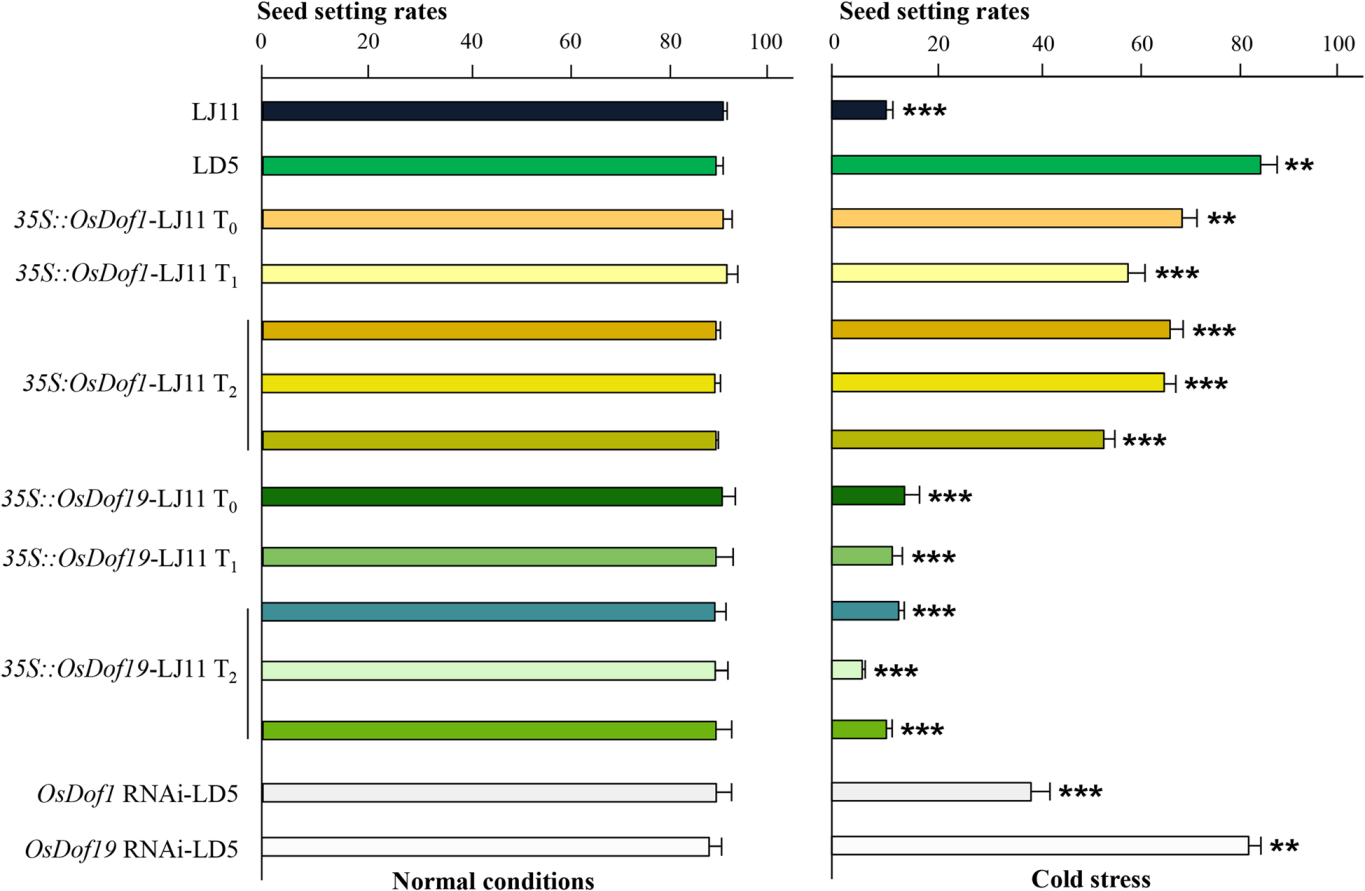
*OsDof1* contributes to cold tolerance in LD5. Seed setting rates of non-transgenic (LD5 and LJ11) and transgenic lines under normal conditions and cold treatment were estimated. Data are shown as mean ± standard deviation of three plants for each line and under a specific condition (Supplemental Table 4). Asterisks indicate significant differences in seed setting rates between control and cold treatment conditions (Student’s *t*-test; *P < 0.05; **P <0.01; ***P<0.001)

The performance of non-transgenic and transgenic LD5 and LJ11 lines in terms of seed setting rates was investigated. Under normal conditions, *OsDof1* and *OsDof19* overexpression LJ11 lines and *OsDof1* and *OsDof19* RNAi LD5 lines exhibited comparable seed setting rates (Fig. 6 and Supplemental Table 4). Under cold treatment, the seed setting rate of LJ11 was 9.58 % compared with 83.7 % for LD5. The *35 S::OsDof1* LJ11 overexpression lines showed a dramatic increase in seed setting rates (T_0_ 71.62 ± 4.46 %, T_1_ 58.43 ± 0.58 %, and T_2_ 62.50 ± 4.78 %) compared with LJ11. However, *35 S::OsDof19* LJ11 showed a similar performance to LJ11 under cold stress. In addition, the *OsDof19* LD5 RNAi line and LD5 exhibited similar seed setting rates, suggesting *OsDof19* played no crucial role in the cold tolerance of LD5. In contrast, the *OsDof1* RNAi line demonstrated a much lower seed setting rate compared with LD5 during cold stress.

Using qRT-PCR, we selected six T_1_ generation plants with the highest expression levels for further study: *35 S::OsDof1* in LJ11: #3, #12, and #15; and *35 S::OsDof19* in LJ11: #9, #18, and #21 (Supplemental Fig. 3 A). We examined the seed setting rates of 20 T_2_ generation plants from the six selected T1 plants and observed a positive correlation between the seed setting rates under cold treatment and the expression of *OsDof1* (Supplemental Fig. 3B and Supplemental Table 5).

## Discussion

The first successful attempt of *Dof* gene isolation from maize was reported in 1995 [49]. The protein product of *Dof* was suspected to bind to the cauliflower mosaic virus 35 S (CaMV35S) promoter [35, 49]. Later studies revealed that DOF proteins contain a highly conserved DNA-binding domain, which forms a single zinc finger motif with four cysteine residues. The *Dof* gene family has been described as a plant-specific TF gene family and have been identified in several plants including Arabidopsis, Chinese cabbage, sorghum, and wheat. *Dof* genes have been widely investigated in a number of monocots [45–47]. In this study, we identified 30 *OsDofs* of two subspecies of rice, *indica* and *japonica*, from publicly accessible genome sequences. We constructed a joint phylogenetic tree based on DOFs from four monocots (rice, barley, wheat, and stiff brome) (Fig. 2). We identified putative orthologues among species as well as paralogues, and potential gene duplications were observed in rice. Based on the highly conserved DNA-binding domains and structural diversities revealed from phylogenetic analyses, the DOF families were reclassified into several subfamilies.

DOFs play a role in each stage of plant growth, including seed germination, vegetative growth, and flowering [25]. In Arabidopsis, DAG1 and DAG2 affect seed germination [30]. PBF in maize and its orthologues in barley and wheat were suggested to regulate seed development [31, 50, 51]. In maize, DOF1 and DOF2 regulate multiple genes involved in carbon metabolism, including a gene encoding phosphoenolpyruvate carboxylase [52]. DOFs have also been shown to function in plants defense and stress responses. Overexpression of *Dof1* in upland cotton (*GhDof1*) was shown to alter seed oil composition and improve tolerance to salt and cold stress [40]. Most *Dof* genes in apple show changes in expression in response to external treatments, such as osmotic stress, salt, cold, and ABA [53]. A recent study reported that the *Dof* gene is regulated in response to drought and salt treatment in rose plants [43].

To identify the *cis*-regulatory elements within the *Dof* promoter region, sequences (1.5 kb in length) upstream from the start codon of *OsDofs* were screened against the PLACE database [42]. More than 60 *cis*-regulatory elements were identified, with suggested regulatory roles in light responses, tissue-specific expression, phytohormone signaling, and stress tolerance [42]. These diverse functions of *OsDofs* have been experimentally documented. For example, overexpression of *OsDof2* promoted not only nutrient ion uptake and accumulation but also flowering in rice [54]. Constitutive expression of *OsDof4* accelerated flowering under both long-day and short-day conditions in rice [55].

Most *OsDofs* have been found to be responsive to ABA, salt, and PEG treatments in rice seedlings [42]. *OsDof15*, which regulates primary root elongation, is repressed by salt stress [56]. Whether *OsDofs* might participate in cold tolerance of rice plants has remained unknown. Therefore, in this study, we explored the potential cold-responsiveness of *Dofs* in rice. *OsDof1* and *OsDof19* were upregulated after cold treatment in the cold-resistant cultivar LD5 (Supplemental Fig. 2). We further validated the role of *OsDof1* in cold tolerance using non-transgenic and transgenic lines under cold stress (Fig. 6). Overexpression of *OsDof1* in the cold-sensitive cultivar LJ11 led to a much higher seed setting rate than that of LJ11 under cold stress. However, the precise role of *OsDof1* in this outcome remains elusive.

Cold stress at early reproductive stage, especially the booting stage, can prove deadly to rice plants [8, 9]. Colder temperatures can deter pollen development, leading to plant infertility [57, 58]. In some studies, cold treatment (15 °C during day and 10 °C at night) caused infertility up to 90 % and loss of grain quality in rice [59, 60]. Our analysis of seed setting rates in the experimental lines suggest that OsDOF1 might contribute to improved performance of rice under cold conditions. The development of male gametophyte in LJ11 and the *35 S::OsDof1* transgenic line upon cold stress, as well as the potential role of OsDOF1 in reproductive development under cold stress, deserve further investigation to reveal the function of *OsDof1* in cold response.

## Conclusions

In this study, two *japonica* cultivars, LD5 and LJ11, which are cold-resistant and cold-sensitive lines, respectively, were subjected to genomic analysis to identify *Dof* genes and their potential functions in the plant response during cold stress. A total of 30 *OsDof* genes were identified and classified based on common motifs. Potential roles of OsDOFs in several biological processes in plants, including the regulation of circadian cycle and flowering, phytohormone biosynthesis and signaling, and stress responses have been reported. Expression profiling of *OsDofs* in both cultivars was carried out, and the results indicated that *OsDof1* and *OsDof19* are cold-inducible genes. A survey of the seed setting rates of *OsDof1* and *OsDof19* overexpression lines and RNAi lines under cold stress indicates that *OsDof1* may contribute to better performance under cold stress in the studied cultivars. Our investigation into the *Dof* gene family identified *OsDof1* as a potential target for genetic breeding of rice with enhanced cold tolerance.

## Materials and methods

### Identification of the rice *Dof* gene family

*Dof* genes were identified from the nucleotide sequences of *japonica* and *indica* available in publicly accessed genome databases. The genome sequence of *japonica* was obtained from the International Rice Genome Sequencing Project (https://rgp.dna.affrc.go.jp) and that of *indica* was obtained from the National Center for Biotechnology Information using the BLAST function. The genome sequences of *japonica* and *indica* were uploaded using profile hidden Markov models (https://www.ebi.ac.uk/Tools/hmmer/) for searching against the Pfam database [61, 62]. Hits with a match to the Dof domain (PF02701) and an E value no greater than 10^–10^ were considered for further study using InterPro and SMART programs (https://www.ebi.ac.uk/interpro/; http://smart.embl-heidelberg.de/).

### Analysis of the genetic structure, chromosomal position, and duplication events of genes

To visualize the genetic structure of genes, GSDS 2.0 [63] was performed using genomic DNA sequences and coding sequences as input. The chromosomal locations of *OsDof*s based on their physical positions were visualized using the Chromosome Map Tool of Oryzabase (https://shigen.nig.ac.jp/rice/oryzabase/). Gene duplications were searched against the plant genome duplication database (PGDD, http://pdgd.njau.edu.cn:8080/) [64]. Two types of duplication events were analyzed (whole genome duplication and tandem duplication).

### Phylogenetic analysis and feature characterization of OsDOFs

Phylogenetic analysis was performed on the 30 identified OsDOFs, and the neighbor-joining method of Clustalx1.87 was used for sequence alignment. A phylogenetic tree was generated in MEGA 6.0 with a bootstrap replicate value set as 1000 and using the maximum likelihood test method [65]. To assess the evolutionary relationships of DOFs across several monocotyledon species, phylogenetic analyses were performed on barley (*H. vulgare*) [45], wheat (*T. aestivum*) [46], stiff brome (*Brachypodium distachyon*) [47], and rice. Protein motifs within OsDOFs were identified using the MEME suite (http://meme-suite.org/db/motifs) using the following parameters: discovery mode as classic; sequence as DNA, RNA, or protein; motif distribution as zero or one occurrence per sequence; and number of motifs as 30.

### Growth and cold treatment of rice plants

Two *japonica* cultivars, LD5 and LJ11, which are cold-resistant and cold-sensitive lines, respectively, were used in this study. The two cultivars were procured from Heilongjiang Province, Northeast China. LD5 has a high tillering capability and lodging resistance and shows resistance to cold stress. LJ11 is an early-maturing cultivar with a high grain yield and quality, but is sensitive to cold stress. Seeds of LD5 and LJ11 were surface-sterilized using 70 % ethanol for 5 min followed by treatment with 1.5 % sodium hypochlorite for 25 min; seeds were then soaked in deionized water for 12 h and left in the dark for 1 day at 25 °C to germinate. Upon germination, the seedlings were grown under a greenhouse with a light intensity at 400 µmol·m^−2^·s^−1^ and relative humidity of 67.2±3.2 %. The average daily temperature was 22.7±0.3 °C (with an average of 23.7±0.6 °C during the day and 21.6±0.4 °C at night). At the trefoil stage, seedlings were transplanted to pots (diameter, 33 cm; four plants per pot). At the booting stage, the pots were moved into a growth chamber for cold treatment at 15 °C for 14 days. Control (untreated) plants remained in the greenhouse with above-described growth conditions. After cold treatment, the treated plants were then moved to the greenhouse where control experiments were performed. Three pots were included per treatment. The heading stage was recorded for each plant and the seed setting rate was calculated.

### Total RNA isolation, cDNA synthesis, and quantitative real-time-polymerase chain reaction (qRT-PCR)

After a growing period of 50 days at natural conditions, the LD5 and LJ11 rice plants were subjected to cold treatment with or without light for 4 or 16 h. Tissues of root, stem, and leaf (100 mg) were harvested and ground into powder using liquid nitrogen. Total RNA was extracted using Invitrogen™ TRIzol® reagent (Thermo Fisher Scientific, Waltham, MA, USA) following the manufacturer’s protocol. cDNA was synthesized from total RNA and diluted 10 times.

qRT-PCR was performed using a PowerUp^TM^SYBR^TM^ Green Master Mix kit on an Applied Biosystems™ 7500 RT-PCR system (Thermo Fisher Scientific). Primers used in qRT-PCR are listed in Supplemental Table 2. The PCR thermal cycle included denaturation at 95 °C for 10 min followed by 40 amplification cycles (95 °C for 1 min, 60 °C for 1 min, and 72 °C for 1 min). The analysis was repeated with three biological replicates, each with three technical replicates. *OsActin1* (LOC_Os03g50885) was used as an internal standard for qRT-PCR. The 2^−ΔΔCq^ method was used to calculate relative gene expression. Unpaired Student’s t-test was performed to detect statistical significance.

### Generation of transgenic overexpression lines and RNAi lines

Total RNA was extracted from 20-day-old leaves of LD5 and LJ11 plants grown in a greenhouse with a 14 h/10 h light/dark cycle with a light intensity at 200 µmol·m^−2^·s^−1^, relative humidity at 50 % and temperatures of 28 °C during the day and 23 °C at night.

*OsDof1* (1235 bp) and *OsDof19* (1138 bp) were amplified using the primers listed in Supplemental Table 2. For the generation of overexpression lines, *OsDof1* and *OsDof19* were cloned into pCM1307 between *Xba*I and *Bam*H1 restriction sites. For the generation of RNAi lines, four DNA fragments, namely *OsDof1-Y1*, *OsDof1-Y2*, *OsDof19-Y1*, and *OsDof19-Y2*, were amplified using primers listed in Supplemental Tables 2, followed by cloning into a pDS1301 vector.

The constructs were mobilized into an *Agrobacterium* strain, EHA105, for rice transformation as previously described [66]. Empty vectors were used as a negative control. Gene expression was assessed in 15 T_0_ generation plants of each transgenic line, and T_1_ and T_2_ transgenic lines were selected by qRT-PCR as described above.

## Supplementary information

Additional file 1 **Supplemental Fig. 1 **A phylogenetic tree based on DOF proteins from Arabidopsis, barley, wheat, stiff brome, and rice. Full-length protein sequences were aligned using ClustalX, and a joint phylogenetic tree was constructed in MEGA 6.0 using the maximum likelihood method.

Additional file 2 **Supplemental Fig. 2***OsDof1* and *OsDof19* were cold-inducible in LD5 leaves. Expression profiling was performed by qRT-PCR with rice *OsActin1* as an internal standard. Three biological replicates were performed for each sample, and each had three technical replicates. Data are shown as mean ± standard deviation of all replicates for each line and under a specific condition. UN: untreated plants grown under natural conditions; CO: cold-treated plants. Treatment was performed under both light and dark conditions.

Additional file 3 **Supplemental Fig. 3 **Cold tolerance and expression of *OsDof1* in transgenic lines. **A** Expression of *OsDof1* and *OsDof19* in T_1_ generations of overexpression lines (30 plants in total). Three biological replicates per transgenic line were examined, each with three technical replicates. Data are shown as mean ± standard deviation of all replicates of each line. **B** Seed setting rates of T_2_ generation plants from the six selected T_1_ plants (*35 S::OsDof1* in LJ11, #3, #12, and #15; *35 S::OsDof19* in LJ11, #9, #18, and #21) under normal and cold stress conditions. Data are shown as mean ± standard deviation of three plants for each line under each condition (Supplemental Table 5). Asterisks indicate significant differences in seed setting rates between control and cold treatment conditions (Student’s *t*-test; *P < 0.05; **P <0.01; ***P<0.001).

**Additional file 4**

**Additional file 5**

**Additional file 6**

**Additional file 7**

**Additional file 8**

## Data Availability

The 30 rice *Dof* genes investigated in this study were derived from the International Rice Genome Sequencing Project (https://rgp.dna.affrc.go.jp) and National Center for Biotechnology Information (https://www.ncbi.nlm.nih.gov/). The nucleotide sequences and locus information are publicly accessible. The accession numbers with links were included in Supplemental Table 2. The accession numbers and amino acid sequences of DOFs from barley, wheat, stiff brome, and Arabidopsis were obtained from the references (https://static-content.springer.com/esm/art%3A10.1007 %2Fs13258-016-0510-7/MediaObjects/13258_2016_510_MOESM1_ESM.docx; https://static-content.springer.com/esm/art%3A10.1007 %2Fs10142-009-0130-2/MediaObjects/10142_2009_130_MOESM1_ESM.pdf; https://static-content.springer.com/esm/art%3A10.1186 %2F1471-2229-12-202/MediaObjects/12870_2012_1229_MOESM1_ESM.pdf) and the Plant Transcription Factor Database (http://planttfdb.gao-lab.org/family.php?sp=Ath&fam=Dof). Information of gene duplication events were publicly available at the plant genome duplication database (PGDD, http://pdgd.njau.edu.cn:8080/). All data generated from the current study are included in this article and the supplemental files.

## Abbreviations

ABA: Abscisic acid
CaMV35S: Cauliflower mosaic virus 35 S
CDWI: Cold deep-water irrigation
*Dof*: DNA binding with one zinc finger
JTT: Jones-Taylor-Thornton
MAPK: Mitogen-activated protein kinase
MDA: Malondialdehyde
ROS: Reaction oxygen species
TF: Transcription factor
