# Supplementary figures and images for: Genome-wide analysis of Dof transcription factors and their response to cold stress in rice (Oryza sativa L.)
Source: BMC Genomics. 2021 Nov 6;22:800. doi: 10.1186/s12864-021-08104-0 (PMC8572462; doi:10.1186/s12864-021-08104-0)

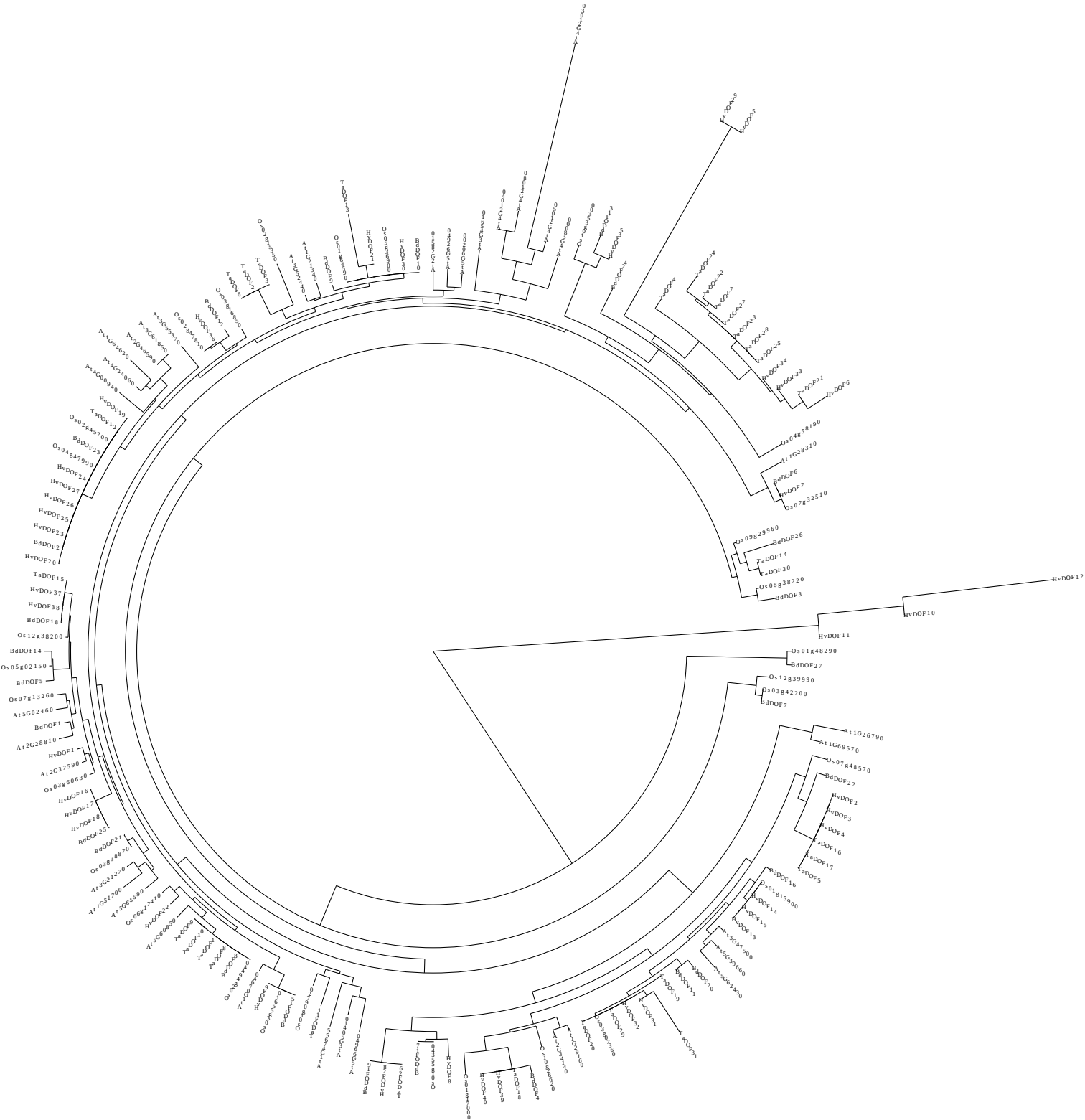

Supplement: Supplementary file 1 — Supplemental Fig. 1 A phylogenetic tree based on DOF proteins from Arabidopsis, barley, wheat, stiff brome, and rice. Full-length protein sequences were aligned using ClustalX, and a joint phylogenetic tree was constructed in MEGA 6.0 using the maximum likelihood method. [file 12864_2021_8104_MOESM1_ESM.pdf]

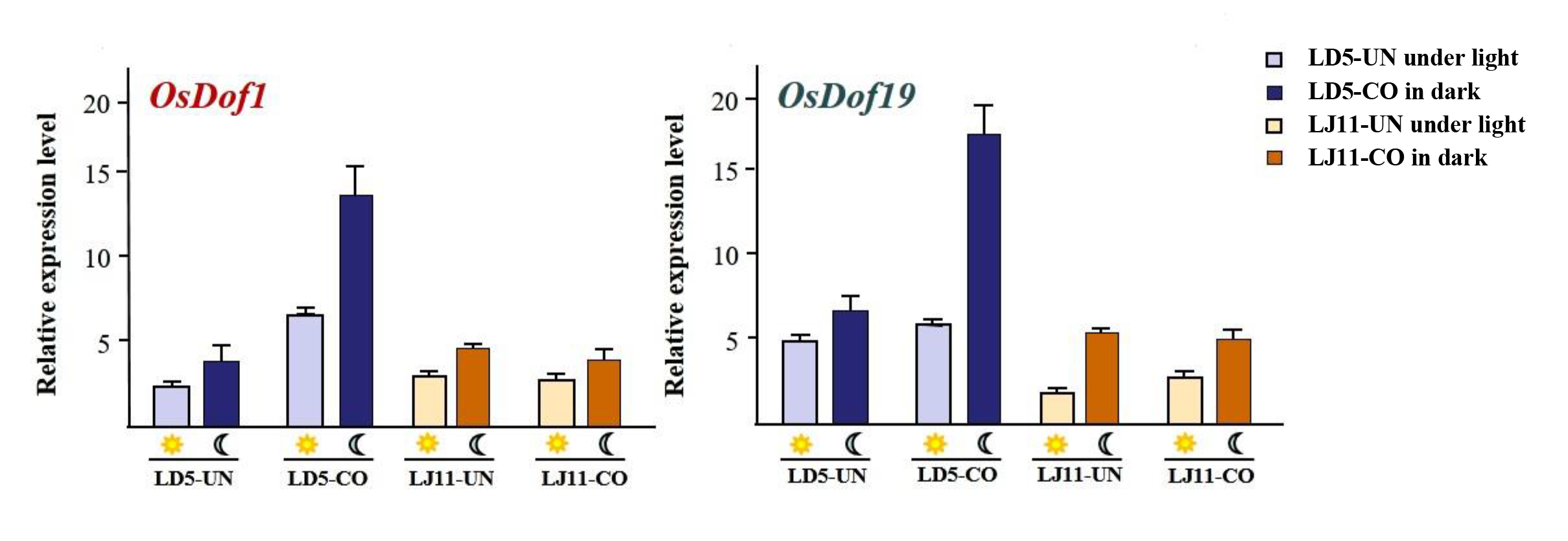

Supplement: Supplementary file 2 — Supplemental Fig. 2OsDof1 and OsDof19 were cold-inducible in LD5 leaves. Expression profiling was performed by qRT-PCR with rice OsActin1 as an internal standard. Three biological replicates were performed for each sample, and each had three technical replicates. Data are shown as mean ± standard deviation of all replicates for each line and under a specific condition. UN: untreated plants grown under natural conditions; CO: cold-treated plants. Treatment was performed under both light and dark conditions. [file 12864_2021_8104_MOESM2_ESM.tif]

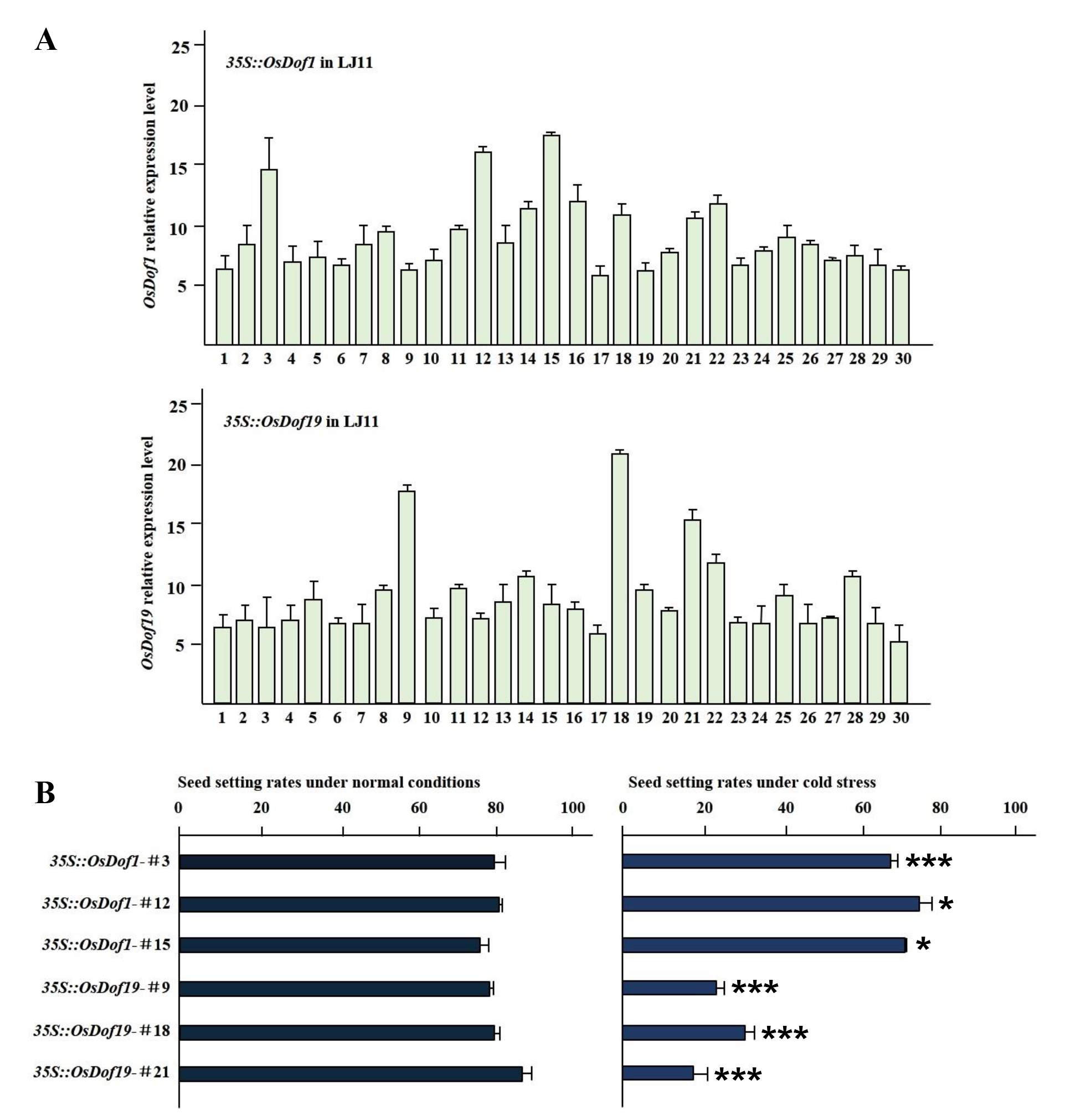

Supplement: Supplementary file 3 — Supplemental Fig. 3 Cold tolerance and expression of OsDof1 in transgenic lines. A Expression of OsDof1 and OsDof19 in T1 generations of overexpression lines (30 plants in total). Three biological replicates per transgenic line were examined, each with three technical replicates. Data are shown as mean ± standard deviation of all replicates of each line. B Seed setting rates of T2 generation plants from the six selected T1 plants (35 S::OsDof1 in LJ11, #3, #12, and #15; 35 S::OsDof19 in LJ11, #9, #18, and #21) under normal and cold stress conditions. Data are shown as mean ± standard deviation of three plants for each line under each condition (Supplemental Table 5). Asterisks indicate significant differences in seed setting rates between control and cold treatment conditions (Student’s t-test; *P < 0.05; **P <0.01; ***P<0.001). [file 12864_2021_8104_MOESM3_ESM.tif]
