# Supplementary material for: Genome-wide analysis of Dof transcription factors and their response to cold stress in rice (Oryza sativa L.)
Source: BMC Genomics. 2021 Nov 6;22:800. doi: 10.1186/s12864-021-08104-0 (PMC8572462; doi:10.1186/s12864-021-08104-0)
Supplement: Supplementary file 4 — Additional file 4 [file 12864_2021_8104_MOESM4_ESM.docx]

**Supplemental Table 1. Common motifs of DOF family proteins**

| **Motif no.** | **Multilevel consensus sequence** |
| --- | --- |
| 1 | EILKCPRCDSMNTKFCYYNNYNLSQPRHFCKTCRRYWTKGGALRNVPVGGGCRKNKR |
| 2 | MEEEKTEKCVWVPKTLRIDDPDEAAKSSIWTTLGIKNDKK |
| 3 | KEEKHHVIETSPVLQANPAALSRSMNFQE |
| 4 | KDPAIKLFGKTIPVP |
| 5 | SMAERARLAKIPLPE |
| 6 | HVMNGVHHPPIKNNGTVLKFGSDAPLCESMASVLNLGEKT |
| 7 | GRLLFPFEDLKQQVSS |
| 8 | SDNNSPTLGKHSRDE |
| 9 | MDTAQWPQEIVVKPIEEIVTNTCPKPP |
| 10 | GYWTGMLGGGSW |
| 11 | DSSRVSQLAPVKTEGNQGLNLSKPYLGIPGNDQY |
| 12 | HIDLALVYAKFLNHH |
| 13 | MVFSSIPVYLD |
| 14 | PHQIPCFPGVPWPYPWNPA |
| 15 | VQLSHLHNILGSQETIANPNFMESKYNIGMLENPRPIDFMDSKFEALVGSSRNYDFMG  NGDLGMVSGLGDMSHHHGLAPNFSDICSPFGMSLDGNSGTFMETCQRLMLPYDQ |
| 16 | HEGQDLNLAFP |
| 17 | DIIGHMPQPQPQLPILPPLHHLGDYNSGDIGLDFGGIQ |
| 18 | FYPVPAYWGCTVP |
| 19 | EKTLKKPD |
| 20 | IERKARPQ |
| 21 | HHHHHHM |
| 22 | GFPLQEFKPTLSFSLDGLGS |
| 23 | HLATTHGGFRHDFPVKRRRCY |
| 24 | KIDQPSVAQMVSVEIQPGNHQPFKNVQENIDFVGSF |

A total of 24 protein motifs were identified within OsDOFs using the MEME suite
